# Construction of key signal regulatory network in metastatic colorectal cancer

**DOI:** 10.18632/oncotarget.23710

**Published:** 2017-12-27

**Authors:** Lu Qi, Yanqing Ding

**Affiliations:** ^1^ Department of Pathology, School of Basic Medical Sciences, Southern Medical University, Guangzhou 510515, China; ^2^ Department of Pathology, Nanfang Hospital, Southern Medical University, Guangzhou 510515, China

**Keywords:** colorectal cancer, regulatory network, mitochondrion, extracellular, tumor microenvironment

## Abstract

There are many stages in the development and metastasis of colorectal cancer (CRC). In this study, we compared the differential expression genes in different stages of metastatic CRC. Then, we screened the continuously up-regulated genes and the continuously down-regulated genes that were associated with the development and metastasis of CRC. After analyzing the intersection of differential expression genes in each stage, we screened the continuously up-regulated genes and deviated genes in the extracellular matrix and the continuously down-regulated genes and deviated genes in the mitochrondia of CRC. Then, we performed gene ontology enrichment analysis of the deviated genes in different phases, and we found that key molecular events occurred in the period extending from stage II to III (early stage of metastasis) of CRC. Furthermore, in this period we found that the chemotaxis of inflammatory cells had decreased in the extracellular matrix. On the other hand, the aerobic respiration had increased in the mitochondrion. Then, we constructed protein-protein interaction network of deviated genes in the extracellular matrix and mitochondrion. We used the network module and hub network to analyze the protein-protein interaction network. The network module analysis showed that the protein complex of VEGFA and CCL7-CCR3 is the key node in the extracellular matrix, while MAPK1 is the key node in the mitochondrion. The hub network analysis showed that the signal transmission chain FN1→SPARC→COL1A1→MMP2 is the key regulatory pathway for extracellular signal transmission. Furthermore, it also showed that CAV1→MAPK3→RAF1→NR3C1→MAPK1→ESR1 is the key regulatory pathway for signal transmission in mitochondrion.

## INTRODUCTION

The development of colorectal cancer (CRC) is a multi-stage process. According to American Joint Committee on Cancer (AJCC), the development of CRC could be roughly classified into four pivotal stages: stage I and stage II is the primary period without metastasis; stage III is the period of lymph node metastasis, and stage IV is the period of distant organ metastasis. During the occurrence of distant organ metastases, the tumor metastasize from the primary focus into the liver, and then it moves from the liver to the lung through the blood stream of the circulatory system. By investigating the molecular events of CRC at each stage, we elucidate its occurrence, development, and metastasis. Presently, many research studies have been conducted to identify the single gene causing CRC. In these studies, scientists have also investigated the mutation and the deletion of classic genes: TP53 [1, 2], APC [3, 4], and SMAD4 [5, 6]. They have found that classic genes are closely related to the development and metastasis of CRC. Several studies have reported about the role of individual gene in promoting the development and metastasis of CRC. For example, HMGB3 [7], RUFY3 [8], FOXK1 [9], TUSC3 [10], AKAP-9 [11], TLE4 [12], FOXC2 [13] genes, etc. promote the development of CRC. Furthermore, it was widely reported that a single gene can inhibit CRC. For instance, MIER3 [14], LASP2 [15], SIRT1 [16], and LZTS1 [17] genes can inhibit the development of CRC. Because the neoplasm develops through a complex process, it cannot be completely elucidated by understanding the changes in a single gene; therefore, the aims of this study are as follows: to elucidate the molecular mechanism of the development of CRC in the midst of each stage generally, and to analyze the links among signal control network during each stage. Presently, the colorectal cancer signal network is mainly comprehended by the following process: the differentially expressed genes of normal tissue were compared with those of tumor tissue, and a signal network [18] constructed by these genes was elucidated. In some cases, a network [19] was formed by combining differentially expressed genes with differentially expressed miRNA. Furthermore, we were able to elucidate network [20] based on available database information, and network [21] formed by combining drug binding target. Nevertheless, these studies failed to elucidate the molecular changes that occur in each phase of CRC; this information is absolutely essential to comprehend the molecular mechanism of the development and metastasis of CRC, and to identify new drug-binding target. After evaluating the expression profile data of CRC and tumor tissue expression at seven different developmental stages of CRC, we screened out the continuously activated genes and continuously suppressed genes at each stage of CRC development, that is, from normal tissue to primary focus to metastases. However, few genes were continuously activated or suppressed for the entire stage of CRC. Genes that were not continuously activated or suppressed during each stage, would best convey the molecular changes of neoplasm during each phase of CRC. Therefore, this study primarily analyzed these genes for elaborating the molecular events of each stage of CRC. Furthermore, the interaction network of CRC proteins was implemented in this study. For this purpose, we considered the interaction of the corresponding proteins of these genes, and we marked the molecular events that occurred during each phase of neoplasm development in the network. With this strategy, we elucidated the core signal regulatory network.

## RESULTS

### The analysis of molecular events at each stage of CRC development

By analyzing gene expression changes at each stage of CRC, we identified continuously activated genes and continuously suppressed genes associated with CRC development. Our analysis was based on the data obtained from seven stages: normal, stage I, stage II, stage III, stage IV, liver metastases, and lung metastases. The data indicates that the number of continuously activated genes and continuously suppressed genes decreased with the development and progression of CRC from one stage to the next. Deviating genes could not maintain continuously activated or continuously suppressed status at every stage of CRC. By comprehensively analyzing these deviating genes, we were able to recognize the molecular events underlying the different developmental stages of CRC.

**Figure 1:**
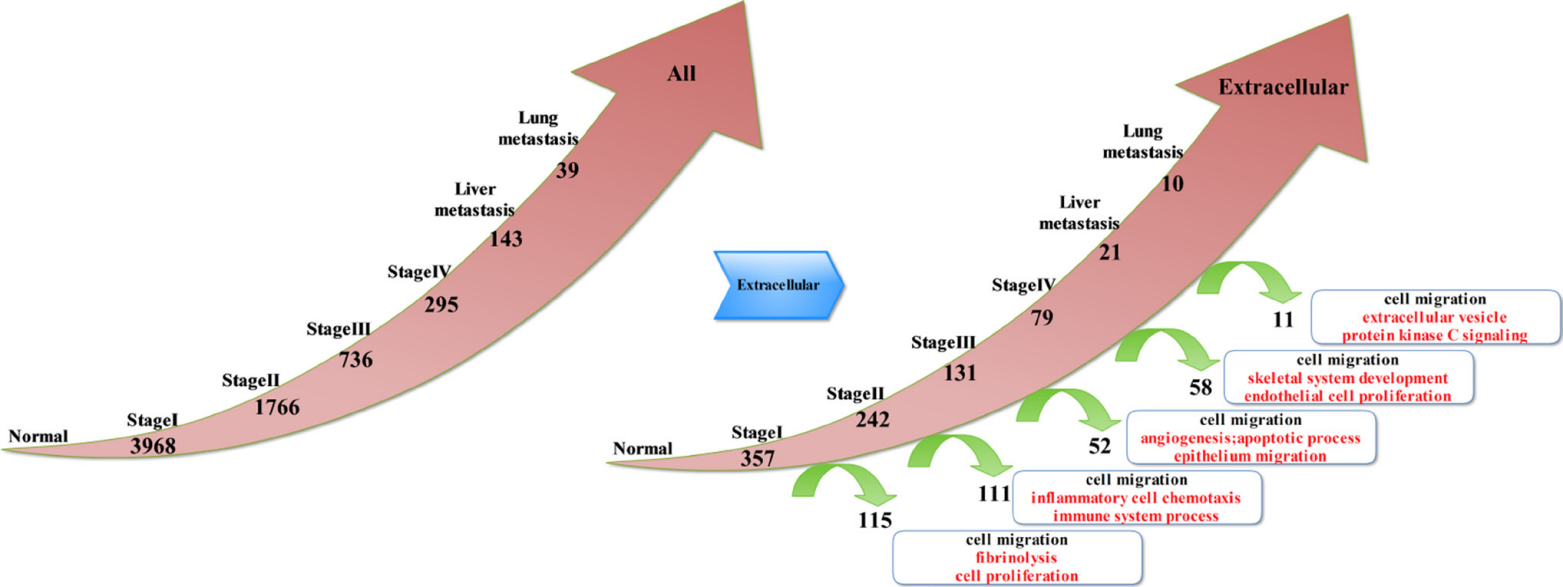
Key molecular events associated with the extracellular deviated genes in different stages of CRC

By analyzing the continuously activated genes (Figure 1), we obtained the following results: compared to the normal tissue, 3968 genes were up-regulated at stage I and 1766 were up-regulated at stage II; there were 736 up-regulated genes at stage III, and there were 295 up-regulated genes at stage IV. Furthermore, 143 genes were up-regulated at liver metastases stage, and 39 genes were up-regulated at lung metastases stage. The analyses results indicate that many of these genes were located in the extracellular matrix; therefore, genes belonging to the extracellular matrix were extracted from the activated genes in each of the above stages of CRC. Thus, we obtained the continuously activated genes and the deviated genes in the extra-cellular phase: compared to the normal tissue, there were 357 up-regulated genes at stage I and 115 deviated genes. Furthermore, 242 genes remained up-regulated at stage II but 111 genes got deviated. There were 131 up-regulated genes at stage III, but 52 genes had deviated. Moreover, 79 genes were up-regulated at stage IV, and 58 genes had deviated. Only 21 genes were up-regulated at liver metastases stage, and 11 genes were deviated. Finally, 10 genes remained up-regulated at lung metastases stage. By analyzing the deviation of genes at each stage, we could comprehend the molecular events at each stage of CRC development. Therefore, we selected the biological process of Gene Ontology to perform enrichment analysis of the genes that deviated from each stage of CRC. Thus, we detected cell migration and movement events at each stage. In addition to the same molecular events, different stages of tumor development also had their own characteristics. At stage I to stage II of CRC, we found that molecular events were mainly cell proliferation and fibrinolysis. At stage II to stage III of CRC, we found that molecular events mainly included chemotaxis and migration of inflammatory cells, and the immune system processes. At stage III to stage IV of CRC, molecular events were mainly vasculogenesis, and apoptosis and epithelial cell migration. In stage IV to liver metastasis, bone morphogenesis and endothelial cell proliferation were the main molecular events. In hepatic metastasis to lung metastasis, the molecular events were primarily extracellular vesicle and protein kinase C signal pathway. As the tumor progressed to late stages, the number of continuously active genes decreased. Consequently, the number of deviated genes decreased proportionately. Thus, the molecular events of late stages reflected changes in the expression of a few genes.

**Figure 2:**
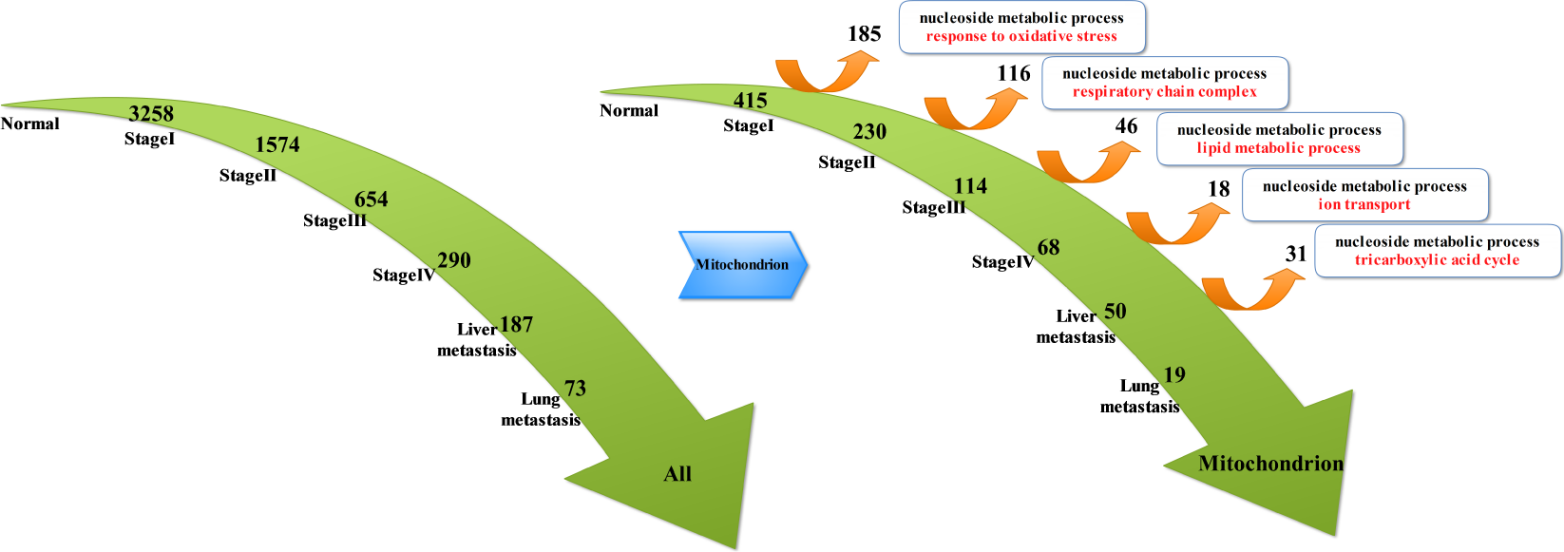
Key molecular events associated with the mitochondrial deviated genes in different stages of CRC

By analyzing continuously suppressed genes (Figure 2), we obtained the following results: compared to the normal tissue, 3258 genes were down-regulated at stage I and 1547 genes were down-regulated at stage II. Moreover, 654 genes were down-regulated at stage III and 290 genes were down-regulated at stage IV. There were 187 down-regulated genes at liver metastases stage, and 73 down-regulated genes were detected at lung metastases stage. Throughout the analysis, we found that most of the genes were located in the mitochondria. Therefore, we extracted genes that belong to the mitochondria from the suppressed genes in each stages of CRC. These genes from the mitochondria were further classified as suppressed genes and deviated genes at each stage of CRC: compared to the normal tissue, there were 415 down-regulated genes at stage I and 185 deviated genes, whereas 230 genes remained down-regulated at stage II but 116 genes had deviated. Moreover, 114 down-regulated genes remained at stage III but 46 genes had deviated. There were 68 down-regulated genes at stage IV and 18 genes had deviated. There were 50 genes remained down-regulated at liver metastases stage but 31 genes had deviated. Finally, Only 19 genes remained down-regulated at lung metastases stage. Since deviated genes of each stage reflected molecular events of each stage of tumor development, we used the biological processes in Gene Ontology to perform enrichment analysis of the genes that deviated from each stage. Thus, we detected nucleoside metabolic process at each stage. Besides the same molecular events, all the stages of tumor development also had their own characteristics: in stage I to stage II of CRC, molecular events mainly elicited oxidative stress response; in stage II to III of CRC, molecular events mainly included respiratory chain components; in the stage III to IV of CRC, molecular events mainly consisted of fatty acid metabolism; from stage IV to liver metastases, molecular events mainly included the transport of ions, especially cationic transport. In liver metastasis to lung metastasis, molecular events mainly included tricarboxylic acid cycle. As the CRC developed and progressed to later stages, there was a decrease in the number of continuously suppressed genes. Consequently, the number of deviated genes also decreased simultaneously; therefore, in the late stages of CRC tumor, the molecular events could only reflect changes in the expression of a few genes.

### Construction of key signal regulatory network of CRC

**Figure 3:**
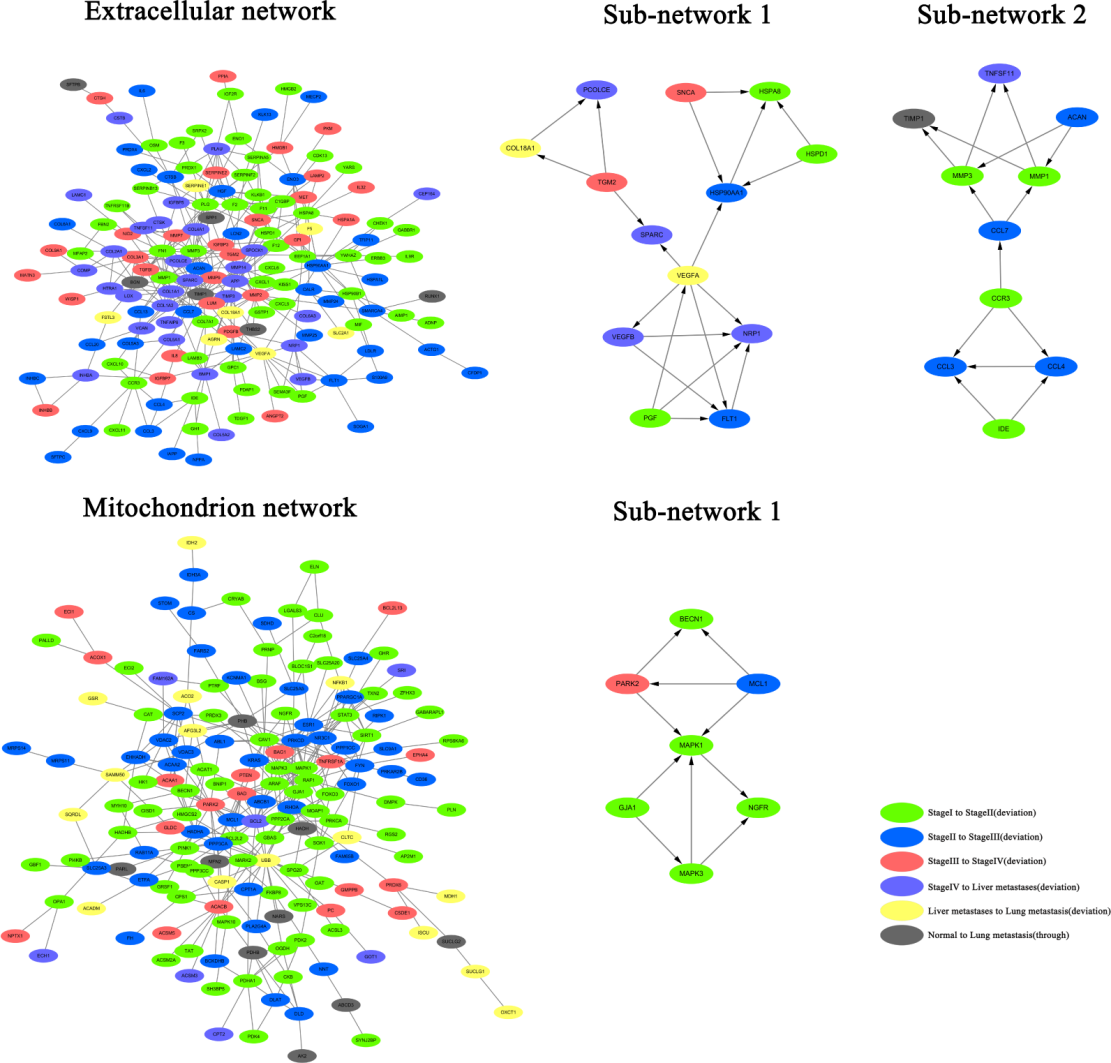
Construction of protein-protein interaction network in the extracellular matrix and mitochrondria, and the analysis of network modules in CRC

The above-mentioned analysis confirm that both deviated genes and non-deviated genes participate in different stages of CRC. The protein-protein interaction network was constructed from the STRING database. The networks of continuously up-regulated genes and continuously down-regulated genes were established independently as they anchored the extracellular matrix and mitochondrion, respectively (Figure 3). First of all, the two network modules were analyzed by MCODE plug-in of Cytoscape software. There are two signal sub-networks in the extracellular matrix; the sub-network 1 consisted of three parts. The key node was VEGFA, which is the deviated gene that progresses from liver metastasis to lung metastasis. In the sub-network 1, the three genes that interacted with VEGFA were the deviated genes that progressed from stage IV to liver metastasis. Furthermore, these three genes were also involved in vascular endothelial proliferation, indicating that VEGFA could be the key gene of CRC metastasis. The sub-network 2 comprised of two parts that had key nodes of protein complexes, namely, CCR3 and CCL7. CCR3 was a chemokine receptor protein, while CCL7 was chemokine ligand protein. CCR3 and CCL7 could together establish connections with the chemotactic factor family and the matrix metalloproteinase family. In particularly, CCL7 directly affected on MMP1 and MMP3, indicating that it was perhaps the key node that connects the chemotaxis of inflammatory cells with cytoplasmic movement. The analysis of mitochondrion indicated that the key signaling network in mitochondrion is governed by the tight interaction between the respiratory chains. Therefore, the node related to respiratory chains was canceled out from the network of mitochondrion. According to the results of module analysis, sub-network 1 was found in the mitochondrion with the key node MAPK1. This indicates that MAPK signal pathway is closely associated with the development of CRC in mitochondrion.

**Figure 4:**
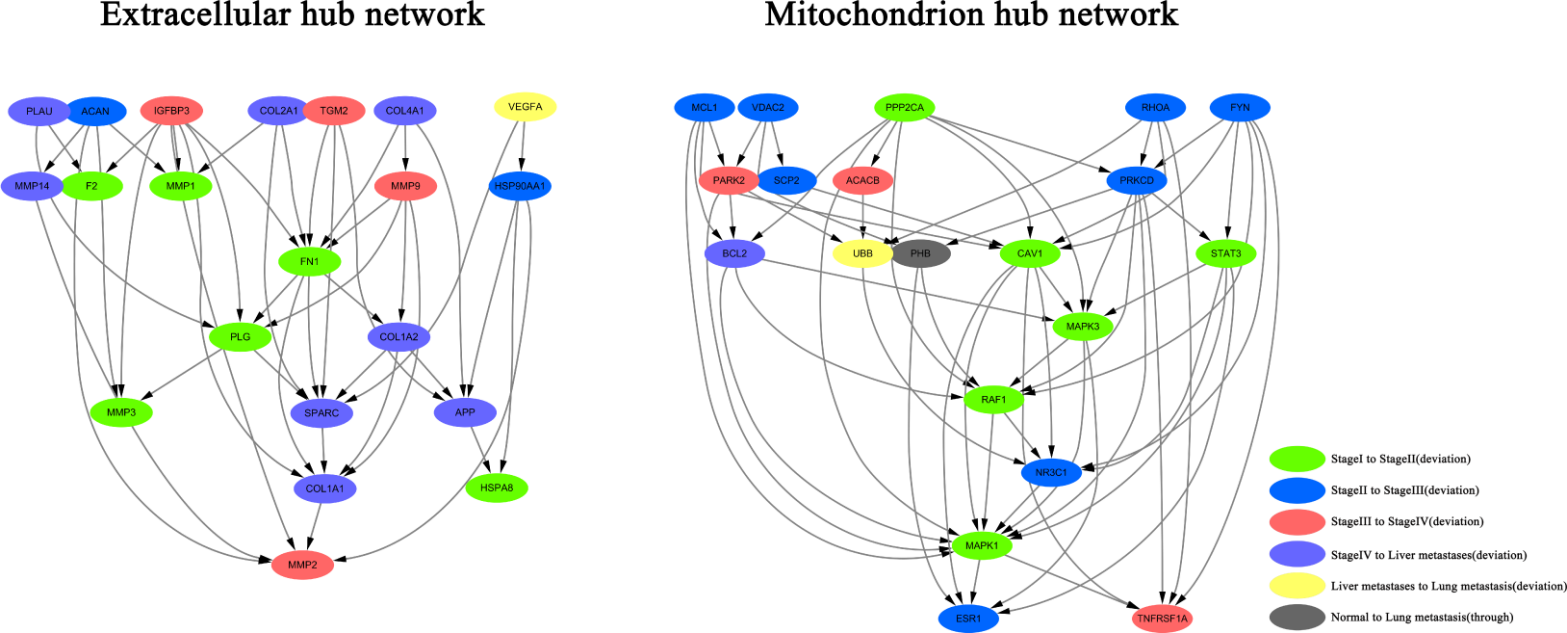
Construction of extracellular and mitochondrial hub network in CRC

Since there were complex signal regulatory networks in the extracellular matrix and mitochondrion, the hub node (the major protein node) had a large interaction range. Therefore, we used the second method to analyze the signal regulatory network in the extracellular matrix and mitochondrion (Figure 4). Finally, we counted the linking numbers of protein node. The results indicated that the highest linking number was 18 in the extracellular matrix. The highest linking proteins are *FN1* and *MMP2*. The highest linking number was 19 in mitochondrion after removing the connection between the protein node and respiratory chains. The highest linking proteins are *CAV1*. In order to control the network size and to clearly identify the key regulatory node, we selected 21 protein nodes from the extracellular matrix and considered them as hub nodes; the linking number of these nodes was in the range of 8 to 18. Furthermore, we selected 20 protein nodes were selected from the mitochondrion and considered them as hub nodes; the linking number of these nodes was in the range of 8 to 19. The hierarchical layout of these hub nodes was constructed by Cytoscape software to understand the hub network in extracellular matrix and mitochondrion. Based on the results of the hub network in the extracellular matrix, we conclude that proteins are mainly present in the constitution of extracellular matrix; therefore, they are closely associated with cytoplasmic movement. The deviated genes were mainly discovered after stage III, indicating the correlation of these genes with tumor metastasis. The four key transfer points in the extracellular matrix were as follows: FN1→SPARC→COL1A1→MMP2. These points may be forming an important signal transmission pathway for protein interaction in extracellular matrix. The linking number was the highest in the hub nodes FN1 and MMP2. In the hub network of mitochondrion, we found that some genes participated in the MAPK signaling pathway, but many of them deviated in stage II and III. The six key transfer points in mitochondrion were as follows: CAV1→MAPK3→RAF1→NR3C1→MAPK1→ESR1. The linking number of the hub node CAV1 was the highest, while the linking number of MAPK1 was slightly less than that of CAV1.

## DISCUSSION

In the carcinogenesis, development, and metastasis of CRC, there are many stages of progression. The key signaling molecule was different in each stage. In this study, we analyzed the expression of continuously up-regulated genes and the continuously down-regulated genes in the seven stages of CRC, which progressed from the normal stage to lung metastasis. We screened the deviated genes. Then, we performed gene ontology enrichment analysis to confirm the participation of these genes in the various biological processes of the extracellular matrix and mitochondrion. After identifying many identical genes in the key molecular events of the same stage, we came across some important phenomena: Some continuously activated genes in the extracellular matrix, such as chemokine ligand gene and chemokine receptor genes participated in the chemotaxis of inflammatory cells. These genes also deviated rapidly from stage II to stage III. however, among these deviated genes, chemokine genes ceased to exist after stage III. Among the continuously down-regulated genes of the mitochondrion, some were associated with the component of respiratory chains; these genes deviated rapidly from stage II to stage III. After stage III, there was a significant decline in the number of deviated genes associated with the components of respiratory chains. In the period extending from stage II to stage III, there was transition from the primary focus to early metastasis (lymphatic metastasis). In this period, the changes in the molecular expression of extracellular matrix and mitochondrion could be the key molecular events of tumor metastasis.

Mitochondrion is the key cell organelle that provides energy in the form of ATP molecules. According to the Warburg effect, the growth rate of cancer cells is much greater than normal cells because they have different pathways for obtaining energy; cancer cells prefer to use glycolysis and not aerobic respiration for producing energy. According to the Pasteur effect, aerobic respiration inhibits anaerobic glycolysis in normal cells. On the other hand, tumor cells showed anti-Pasteur effect. The study found that in the early stage of colorectal cance (stage I and stage II), the respiratory chain components of the mitochondrion showed continuous down-regulation. This indicates that aerobic respiration was inhibited in the early stages of CRC. In the period between stage II and III, there was no inhibition of aerobic respiration in the mitochondrion. On the contrary, aerobic respiration was enhanced in the process of cancer metastasis. In general, aerobic respiration depends on the oxygen supply of blood. Howerver, the oxygen supply in blood decreases when the growth of tumor cells is uncontrolled. To solve this problem, tumor cells undergo anaerobic glycolysis and obtain enough energy for cytomorphosis and cell migration. A better oxygen environment was created by the tumor cells underwent angiogenesis during metastasis, and the process improved the oxygen supply for the cells. This indicates that tumor metastasis is an aerobic process. The components of the extracellular matrix provided the micro-environment for tumor cell growth. In the early stages of CRC, we found that the chemotactic factor can gather inflammatory cells. This indicates that inflammatory cells immensely affect the early stages of CRC. In the period extending from stage II to stage III, the activating effects decreased suddenly. According to previous reports, aerobic respiration was always suppressed in the early stages of cancer; therefore, tumor cells gained energy by anaerobic glycolysis during this period. However, the necrosis, anoxia, and oxidosis of tumor cells promoted the expression and activation of chemotactic factor. Aerobic respiration increased in CRC cells during tumor metastasis. At this stage, oxygen deficiency was rectified by the change in the energy supply mode of tumor cells. Consequently, the activation effect of the chemotactic factor attenuated sharply.

In this study, we determined the changes that occurred in the key signaling molecules during the metastasis of CRC. We found that the chemotaxis of inflammatory cells decreased in the extracellular matrix but the aerobic respiration increased in the mitochondrion during metastasis of CRC. By performing hub network analysis, we found that the signal transfer chain in the extracellular matrix was follows: FN1→SPARC→COL1A1→MMP2. It was probably the key signal regulatory network in the extracellular matrix. We also found that a variety of genes in the extracellular matrix played an important role in tumor metastasis. Furthermore, we found that the signal transfer chain in the mitochondrion was as follows: CAV1→MAPK3→RAF1→NR3C1→MAPK1→ESR1. This chain could be the key signal pathway in the mitochondrion. In conclusion, the changes in the key signal molecules of the extracellular matrix and mitochondrion were key factors for the carcinogenesis, development, an metastasis of CRC. Further studies must investigate the mechanism through which these signaling molecules participate in the carcinogenesis, development, and metastasis of CRC.

## MATERIALS AND METHODS

### Data sources

To analyze the changes in the key signal molecules causing the progression of CRC, we used the GEO database [22, 23] to obtain the data set of gene expression profile of CRC (No. GSE41258). The data set GSE41258 includes 54 cases of normal colorectal tissues, 186 cases of CRC with primary focus (28 cases in stage I, 50 cases in stage II, 49 cases in stage III, 58 cases in stage IV, and 1 case without tumor stage information), 47 cases of CRC with liver metastasis, and 20 cases with lung metastasis.

### Screening of continuously upregulated and downregulated genes during the progression of CRC

**Figure 5:**
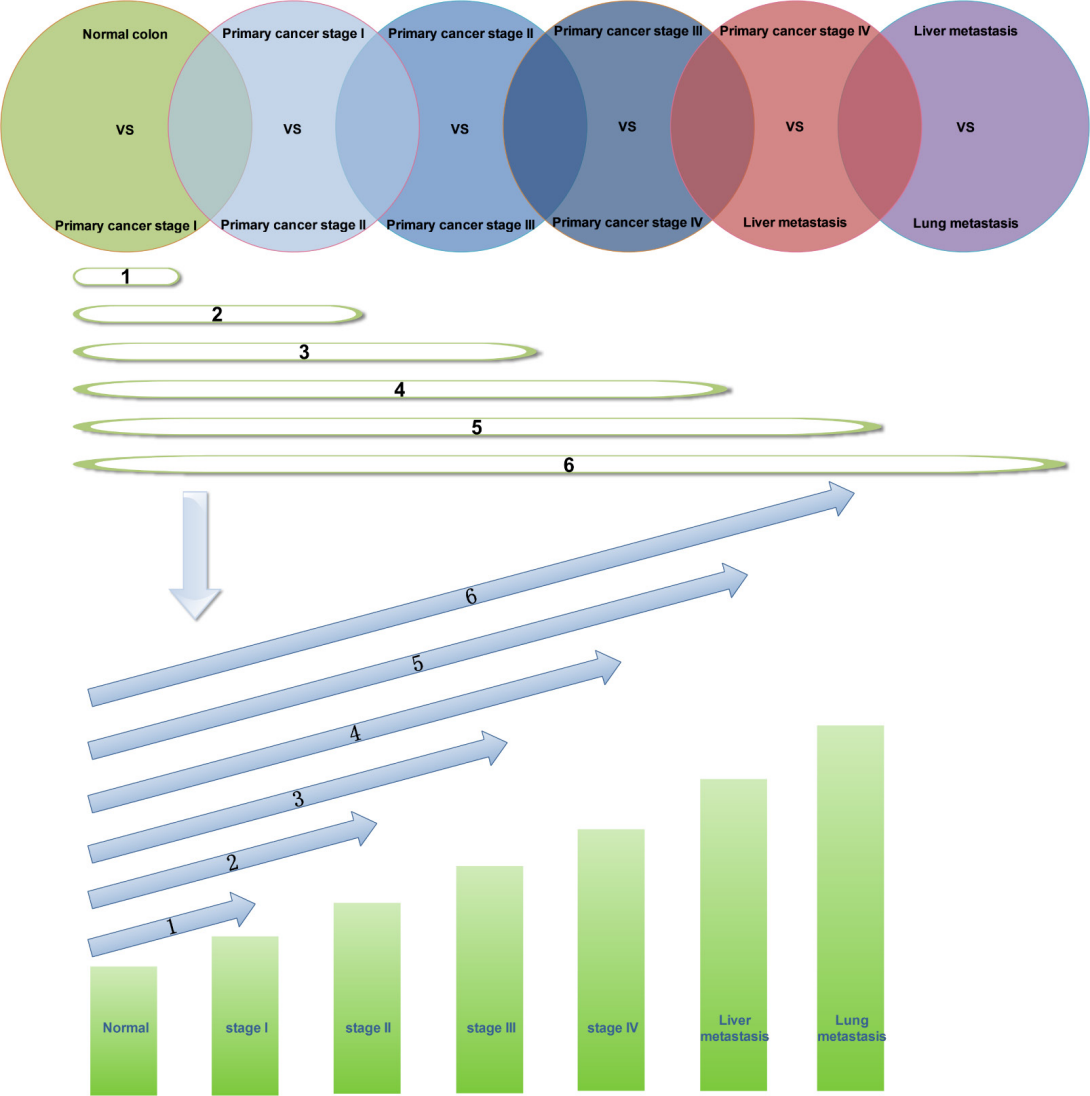
Screening of genes showing continuous upregulation in different stages of CRC

During the progression of CRC, we screened the continuously upregulated and downregulated genes to determine the changes in these key signal molecules. In this study, GSE41258 data were divided in seven groups: normal, stage I, stage II, stage III, stage IV, liver metastasis, and lung metastasis. We screened the differential expression genes and performed paired comparison of various groups: stage I vs normal, stage II vs stage I, stage III vs stage II, stage IV vs stage III, liver metastasis vs stage IV, and lung metastasis vs liver metastasis. The differential expression genes were screened by using Genespring software. Then, one way ANOVA was used for performing statistical analysis. Asymptotic method was used for calculating *p*-value. Bonferroni FWER method was used for performing multiple testing correction [24]. We selected only the differential expression genes whose *p*-value was less than 0.05. Folder change of differential gene was set to 1 time. A significant difference was found between the gene expression of normal tissues and CRC tissues, but only a slight difference was observed in the differential expression genes during the different stages of CRC. When folder change of differential gene was set to 2 time, we could not perform the screening of differential expression genes in different stages of CRC. Bonferroni FWER was used to adjust the *p*-value reliability. There was intersection of the six pairs of differential expression genes, which indicated the progression of CRC. We screened genes that showed continuous upregulation and downregulation. As shown in Figure 5, we observed intersection in the six pairs of differential expression genes; therefore, we screened the genes that showed continuous upregulation in tissues of normal, stage I, stage II, stage III, stage IV, liver metastasis, and lung metastasis. When we observed intersection in only two pairs of differential expression genes, we screened genes showing continuous upregulation only in tissues of normal, stage I, and stage II samples. Thus, we identified genes showing continuous upregulation and downregulation at different stages of CRC.

### Screening and enrichment analysis of key signal molecules in different stages of CRC

After identifying the genes that showed continuous upregulation and downregulation in different stages of CRC, we used ToppGene Suite [25] to perform Gene Ontology [26] enrichment analysis. The enrichment results indicated that continuously upregulated genes had higher scores in extracellular matrix, while continuously downregulated genes had higher scores in mitochondrion. Therefore, the extracellular matrix and extracellular space genes were screened among genes showing continuous upregulation. Likewise, the mitochondrial genes were screened among genes showing continuous downregulation. In different stages of CRC, there were genes for containment; however, the number of such genes decreased with the develpoment and metastasis of CRC. To determine the changes that occurred in key signal molecules in different stages of CRC, we screened the genes that showed deviation. Then, we used ToppGene Suite for performing Gene Ontology enrichment analysis. For example, among the extracellular genes with upregulated expression, 357 genes had upregulated expressions in stage I. Moreover, 242 genes showed continuous upregulated expressions in stage II. In total, 115 genes showed deviation.

### Construction of key signal regulatory network in CRC

We screened both deviated genes and genes that showed no deviation. The STRING [27, 28] database was used to construct a protein-protein interaction network. For ensuring high levels of accuracy, we selected the method of protein connection only after experimental verification. Then, the protein nodes of network were set as input genes. In the first method, we performed the following steps: we analyzed the obtained network using Cytoscape [29, 30] MCODE plug-in [31]. Then, we calculated the existed network modules. Finally, we screened the network module with higher scores and marked the protein nodes in different stages of CRC. In the second method, we performed the following steps: we analyzed the obtained network by screening genes that had more connection numbers (hub nodes). Then, we used Cytoscape for determining the hierarchical layout, and we finally constructed the cellular signaling network from the hub nodes of extracellular and mitochrondial genes. With these two methods, we determined the effect of protein nodes on the key signal regulatory network of CRC. In addition, we also elucidated the molecular mechanism through which deviated genes bring about the progression of CRC.
